# A network-guided protocol to discover susceptibility genes in genome-wide association studies using stability selection

**DOI:** 10.1016/j.xpro.2022.101998

**Published:** 2023-01-06

**Authors:** Héctor Climente-González, Chloé-Agathe Azencott, Makoto Yamada

**Affiliations:** 1 RIKEN Center for Advanced Intelligence Project, Chuo-ku, Tokyo 103-0027, Japan; 2 Centre for Computational Biology (CBIO), MINES ParisTech, PSL Research University, 75005 Paris, France; 3 Institut Curie, PSL Research University, 75005 Paris, France; 4 INSERM, U900, 75005 Paris, France; 5 Department of Intelligence Science and Technology, Graduate School of Informatics, Kyoto University, Sakyo-ku, Kyoto 606-8501, Japan; 6 Machine Learning and Data Science Unit, Okinawa Institute of Science and Technology Graduate University, Onna-son, Okinawa 904-0495, Japan

**Keywords:** Bioinformatics, Genetics, Genomics

## Abstract

We present a network-based protocol to discover susceptibility genes in case-control genome-wide association studies (GWASs). In short, this protocol looks for biomarkers that are informative of disease status and interconnected in an underlying biological network. This boosts discovery and interpretability. Moreover, the protocol tackles the instability of network methods, producing a stable set of genes most likely to replicate in external cohorts. To apply the procedure to a provided GWAS dataset, install the required software and execute our command-line tool.

For complete details on the use and execution of this protocol, please refer to Climente-González et al.^1^

## Highlights

• The protocol finds genes that are functionally related and associated with a phenotype
• Statistical association is measured on a GWAS, co-function, on a gene-gene network
• The set of genes is highly interpretable and likely to replicate on another cohort
• To run it, simply install the software, prepare the GWAS data, and execute one command

## Abstract (editor highlights)

Publisher’s note: Undertaking any experimental protocol requires adherence to local institutional guidelines for laboratory safety and ethics.

## Graphical abstract

**Figure:**
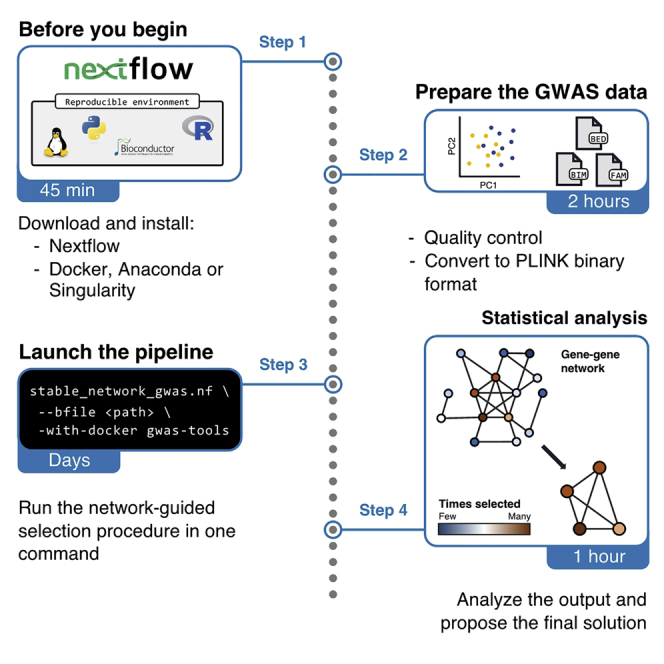

## Before you begin

### Install gwas-tools and its prerequisites

**Timing: 1 h**

The presented protocol is part of gwas-tools (GitHub: https://github.com/hclimente/gwas-tools), a collection of pipelines to handle and analyze GWAS datasets. The first step is installing this collection and all its prerequisites.

***Note:*** The provided pipeline can run on most computational frameworks. However, it could take weeks on a desktop computer (materials and equipment). Hence, we recommend running it on a powerful server, a high-performance cluster, or the cloud.

1. Install a Unix shell (like Bash or Zsh). This is available in most operative systems like Linux distributions (e.g., via the Terminal in Ubuntu), macOS (via the Terminal), or Windows (by installing the Windows Subsystem for Linux).
2. Install git.

***Note:***Git is usually bundled with most operative systems. You can verify that it is already available by executing from the shell:

> command -v git

If available, this command should return the path in which the git executable lives.A

3. Use git to obtain a copy of the gwas-tools repository:

> git clone--depth 1git@github.com:hclimente/gwas-tools.git

4. Include the directory containing the pipelines in your execution path:

> export PATH=$PATH:$PWD/gwas-tools/bin

***Note:*** This command needs to be run in every new session which intends to use gwas-tools (e.g., by adding it to the .bashrc file if working on Bash).

5. All gwas-tools workflows are written in Nextflow,^7^ a platform to handle scientific workflows in a platform-agnostic manner.
  a. Install Nextflow following its official documentation.
  b. Configure it by creating a file called nextflow.config inside the directory from which the pipeline will run. At a minimum, it should read:

> docker.enabled = true

> process.executor = 'local'

***Note:*** The process.executor must match your computing platform. By setting it to local, Nextflow will perform the computations in the same computer in which Nextflow is launched. However, by altering this value, Nextflow can handle common platforms to launch computations remotely, like SGE, SLURM or AWS (see the complete list in Nextflow’s documentation).

6. Docker provides a way to share computational environments. All the pipeline’s dependencies are included in a Docker image available on the Docker Hub: hclimente/gwas-tools. Install Docker following its official documentation.

**Alternative:** If you cannot use Docker but frameworks like Conda or Singularity are an option, refer to Problem 1 in the Troubleshooting section.

7. Do a test run by running the following command from the gwas-tools root directory:

> stable_network_gwas.nf \

--bfile test/data/gwas \

--edgelist test/data/edgelist.tsv \

--sigmod_nmax 6 \

--sigmod_maxjump 1 \

-with-docker hclimente/gwas-tools

Check that this command runs without errors and creates the file stable_consensus.tsv in the working directory (among others).

8. Check the stable_consensus.tsv file. It will highlight six genes highly interconnected in an underlying fictional network (specified in test/data/edgelist.tsv) and highly associated to a phenotype (as measured on the fictional GWAS at test/data/gwas.{bed,bim,fam}). Hence, the first seven lines of this file should read:

gene n_selected methods

ADM2 25 dmgwas(5),heinz(5),lean(5),scones(5),sigmod(5)

CHEK2 25 dmgwas(5),heinz(5),lean(5),scones(5),sigmod(5)

EP300 25 dmgwas(5),heinz(5),lean(5),scones(5),sigmod(5)

FBLN1 25 dmgwas(5),heinz(5),lean(5),scones(5),sigmod(5)

MAPK1 25 dmgwas(5),heinz(5),lean(5),scones(5),sigmod(5)

RBX1 25 dmgwas(5),heinz(5),lean(5),scones(5),sigmod(5)

### Prepare the files used in the analysis

**Timing: 15 min**

Prepare the GWAS dataset on which we will find genes associated with the phenotype. Optionally, prepare also the files describing the gene-gene interaction network and the linkage disequilibrium patterns in the population.

9. Prepare the case-control GWAS dataset on which to conduct the biomarker discovery procedure. It must be in PLINK binary format (bed, fam, and bim).

***Note:*** If it is not in PLINK binary format already, you can probably make the conversion using PLINK 1.9^3^ using the -make-bed flag. (PLINK needs to be installed separately.) For instance, if your data is in PLINK text format (ped and map), the following command will take input.ped and input.map and produce output.bed, output.fam, and output.bim:

> plink -file input -make-bed -out output

***Note:*** The pipeline does not perform quality control steps (e.g., imputation, sample and SNP filtering, population structure stratification). If required, perform them *a priori*.

***Note:*** If you only have access to summary GWAS statistics, refer to Problem 2 in the troubleshooting section.

***Optional:*** Prepare a file describing the gene-gene interaction network. It must be a tab-separated table enumerating the edges by their terminal nodes. The two columns must be named “gene1” and “gene2”. Since the methods assume that the network is undirected, the order of the genes does not matter.

***Note:*** If this file is not provided, the pipeline will query HINT^8^ for all human protein-protein interactions obtained via high-throughput experiments.

***Optional:*** Prepare a GWAS dataset to be used as the reference for linkage disequilibrium patterns. It needs to be in PLINK binary format (bed, fam, and bim).

***Note:*** If this dataset is not provided, the pipeline will use the control samples in the GWAS dataset.

***Note:*** One option are ancestry-matched genotypes from the Phase 3 of the 1000 Genome Project.^9^ They can be downloaded in PLINK binary format from the VEGAS2 page (section Offline version).

**CRITICAL:** Make sure the samples in this dataset have similar ancestry to those in the dataset from step 9 (e.g., they both target the same population). Failure to do so might produce artifactual results. In case of doubt, do not provide this file.

## Key resources table

**Table:** 

REAGENT or RESOURCE	SOURCE	IDENTIFIER
**Software and algorithms**		
Docker	https://www.docker.com	https://www.docker.com
gwas-tools	GitHub	https://github.com/hclimente/gwas-tools
gwas-tools Docker image	Docker Hub	https://hub.docker.com/r/hclimente/gwas-tools
Nextflow	Di Tommaso et al. (2017)^7^	https://nextflow.io
**Other**		
Case-control GWAS dataset	Provided by the user	N/A
(Optional) Gene-gene interaction network	Provided by the user	N/A
(Optional) Ancestry-matched GWAS dataset from the general population	Provided by the user	N/A

## Materials and equipment

The workflow presented below can run on most computational environments, efficiently using the available resources if Nextflow is configured appropriately (see step 5.b in install gwas-tools and its prerequisites). Since many steps are independent, they will run in parallel whenever possible. Hence, more available CPUs will result in lower computing time. Ultimately, the computational time and memory requirements will depend on the size of the GWAS dataset and the complexity of the network. For instance, the analysis of Climente-González et al. (2021)^1^ took 378.1 CPU hours (around 15.8 days) on a CentOS 7 Linux server. By allowing up to 60 processes to run in parallel, the protocol took 68.8 actual hours (around 2.8 days). In terms of memory, most methods used less than 16GB of RAM.

## Step-by-step method details

**Figure 1:**
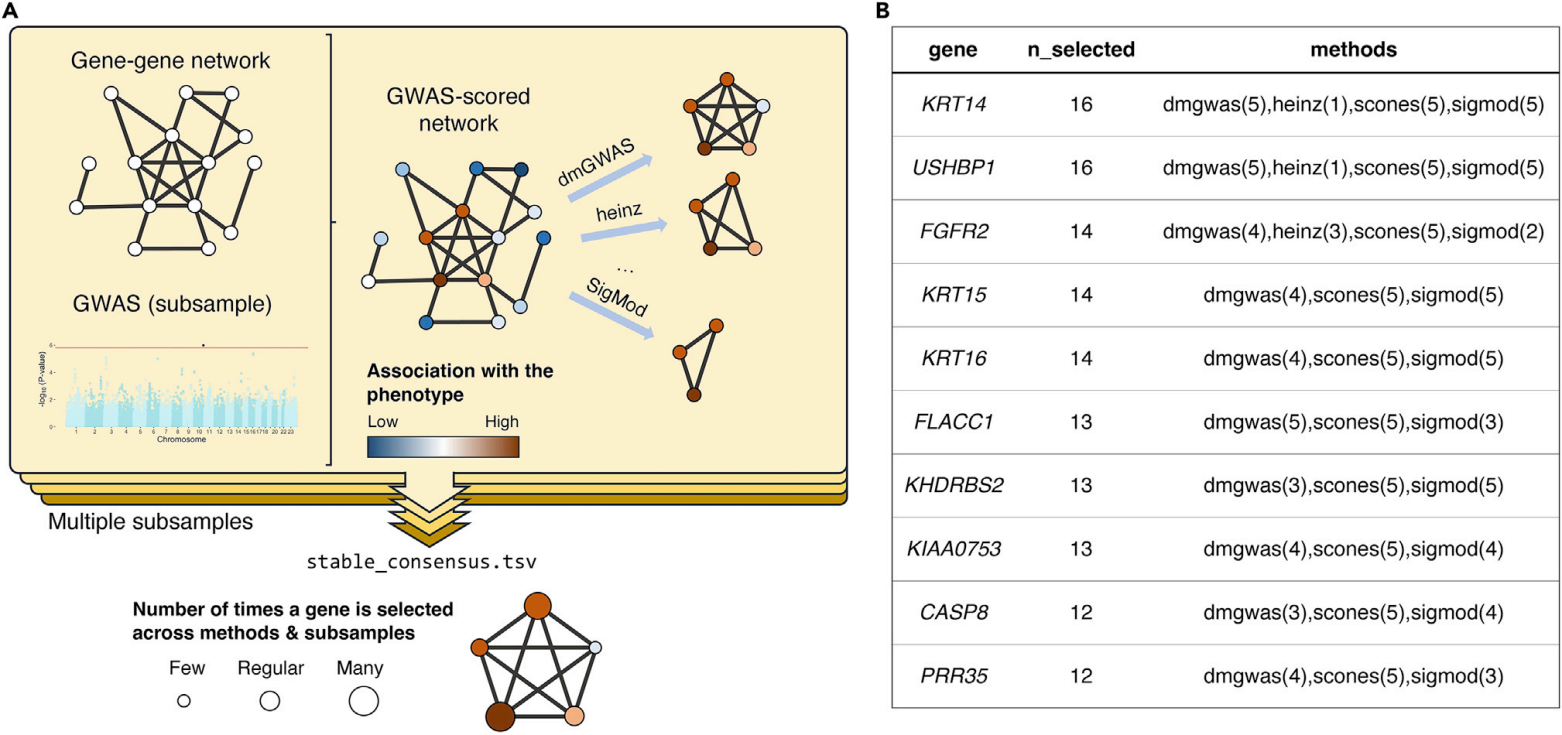
Overview of the pipeline stable_network_gwas.nf and its output (A) Visual depiction of the pipeline. The Manhattan plot comes from in Climente-González et al. (2021).^1^ (B) Top 10 lines from the stable_consensus.tsv file applied to the data in Climente-González et al. (2021).^1^

We have encapsulated the complex pipeline from Climente-González et al. (2021)^1^ into a single command (see run the pipeline and Figure 1A). The pipeline performs the following steps:

Subsample the input GWAS dataset multiple times (five, by default) without replacement. Each equally sized subsample is analyzed independently, and only the final results are aggregated in the last step. The rationale is that the common part of the solutions obtained independently captures something true about the data. In contrast, the differing parts result from the sampling procedure and idiosyncrasies of the algorithm.

Compute a chi-squared test of association between each SNP and the phenotype using PLINK 1.9.^10^

Compute a gene-level association score based on the association at the SNP level using VEGAS2.^11^

Use five algorithms to find important genes in a biological network: dmGWAS,^2^ heinz,^3^ LEAN,^4^ SConES^5^^,^^12^ and SigMod.^6^ SConES uses SNP-level information, while the rest uses gene-level information.

Compile the solutions of the different methods on the multiple subsamples.

### Run the pipeline

**Timing: days (see**materials and equipment**)**

Run the pipeline on your dataset to discover the set of associated biomarkers.

1. Initialize the Docker daemon.
2. Run gwas-tools’ stable_network_gwas.nf:

> stable_network_gwas.nf \

--bfile <path> \

--with-docker gwas-tools

The flag --bfile indicates the base name (i.e., the path without the extension) of the GWAS files in PLINK binary format (bed, fam and bim). For instance, if the three files are located in subdir/gwas_data.{bed,bim,fam}, it should take the value subdir/gwas_data.

***Note:*** The complete list of possible arguments are available in Table S1. For instance, you can include a file specifying the gene-gene interaction network (step 10) via the --edgelist flag. Or a file containing the linkage disequilibrium reference panel (step 11) via the --vegas2_bfile_ld_controls flag. By default, their value is the same as in Climente-González et al.^1^

***Note:*** If you need to re-run the pipeline, refer to Problem 3 in the troubleshooting section.

***Note:*** Pipeline arguments like ‘--bfile’ (full list in Table S1) need to be preceded by two dashes (‘--’), while Nextflow arguments like ‘-with-docker’ or ‘-resume’ (full list in Nextflow’s documentation) are preceded by only one dash (‘-’).

## Expected outcomes

The step 2 from Run the pipeline produces a file named stable_consensus.tsv (see an example in Figure 1B). This file summarizes the networks obtained by the different methods on the different subsamples of the data. It contains three columns: gene, containing the official symbol of each gene; n_selected, containing the number of times each gene was part of the solution subnetwork from any method and subsample; and methods, containing the list of runs which selected each gene. For instance, “dmgwas (3), scones (1)” indicates that the gene was selected four times in total: by dmGWAS in three of the subsamples, and by SConES in one. The pipeline will also copy the outputs of the different algorithms on the different subsamples in the working directory.

***Note:*** If the results differ significantly from what you expected, refer to Problem 4 in the troubleshooting section.

## Quantification and statistical analysis

**Timing: 1 h**

Often stable_consensus.tsv requires a curation step to obtain a final subnetwork, which depends heavily on the desired outcome and the nature of the data. For reference, we include here the steps followed by Climente-González et al. (2021)^1^ to find a stability-based consensus network:

1. Study how often each gene was selected e.g., using a histogram. In Climente-González et al. (2021),^1^ this produced a long-tailed histogram in which a few genes were often selected, while the rest were only present in a few runs.
2. Based on this inspection, decide on a threshold to determine which genes were selected “often enough.” For instance, Climente-González et al. (2021)^1^ only considered the top 1% most selected genes.
3. From the original gene-gene interaction network, take the subnetwork consisting only of the genes selected more often than the threshold chosen in step 2. Include all the edges connecting these nodes in the original network in this subnetwork.

## Limitations

The current pipeline contains several compromises and arbitrary thresholds. For instance, it cannot handle continuous phenotypes and it only implements five of the many network-guided discovery methods in the literature. Nonetheless, since the pipeline is open-source and modular, existing steps can be easily adjusted, and new steps can be added to account for these and other use cases.

## Troubleshooting

### Problem 1

I do not have permission to run Docker on my computing platform (install gwas-tools and its prerequisites, step 5).

### Potential solution

In some environments where Docker is not allowed, Singularity (https://sylabs.io/singularity/) is. If that is the case:

• Install Singularity following its official documentation.
• In step 5.b, replace docker.enabled = true by singularity.enabled = true in the nextflow.config file.
• Test the pipeline as in step 7, but replacing -with-docker gwas-tools by -with-singularity gwas-tools.

Alternatively, you can install all the dependencies in a Conda environment called gwas-tools. To do that:

• Install Miniconda 3 following its official documentation.
• Install mamba in the base environment:

> conda install mamba -n base -c conda-forge

• Create the Conda environment. To do that:
  ○ Change your working directory to the gwas-tools root directory.
  ○ Run the following command:

> make conda

• Load the newly created environment.

> conda activate gwas-tools

• Test the pipeline as in step 7, but removing -with-docker gwas-tools.

### Problem 2

I only have summary statistics for a GWAS, but I do not have access to the genotypes (prepare the files used in the analysis, step 9). This is common when using datasets from the literature.

### Potential solution

Stability selection is an integral part of the protocol, which requires the genotype data to subsample from it. However, four algorithms can be used directly on summary statistics (dmGWAS, Heinz, LEAN and SigMod). We provide an interface to run them on summary statistics on our repository.

### Problem 3

I need to run the pipeline multiple times to explore some hyperparameters (e.g., changing a method’s FDR threshold), but each run takes too long (Run the pipeline, step 2).

### Potential solution

Add the flag -resume. Nextflow automatically caches the intermediate results and tries to reuse them whenever possible. You can find more information in Nextflow’s documentation.

### Problem 4

The selected genes differ significantly from the expected results (expected outcomes). For instance, they are very different from the results of a conventional GWAS.

### Potential solution

Make sure that the SNP coordinates in the GWAS dataset match the genome version specified via the --genome_version flag (Table S1). If a method seems miscalibrated (e.g., it fails to select any gene), try tuning its hyperparameters (Table S1). For guidance, we refer to Climente-González et al. (2021) and to the methods’ respective publications.^1^^,^^2^^,^^3^^,^^4^^,^^5^^,^^6^^,^^13^

### Problem 5

I faced a problem not described here.

### Potential solution

You can open an issue on our GitHub repository (GitHub: https://github.com/hclimente/gwas-tools/issues) or write us an e-mail (hector.climente@riken.jp). Since the code is open source, you can also adapt it to your needs.

## Resource availability

### Lead contact

Further information should be directed to and will be fulfilled by the lead contact, Héctor Climente-González (hector.climente@riken.jp).

### Materials availability

This study did not generate new unique reagents.

## Data Availability

The code of the presented protocol is available on GitHub: https://github.com/hclimente/gwas-tools (>= v1.1.0, Zenodo: https://doi.org/10.5281/zenodo.7395332), under a GPLv3 license. Different licensing terms might apply to the used tools, as you should verify. If you use the results of these tools in your publication, please cite the relevant articles as well.^2^^,^^3^^,^^4^^,^^5^^,^^6^^,^^13^

## Supplemental information

Table S1. Description of the flags of stable_network_gwas.nf, related to step 2 in Run the pipeline
